# A Brief Review of Poly (Butylene Succinate) (PBS) and Its Main Copolymers: Synthesis, Blends, Composites, Biodegradability, and Applications

**DOI:** 10.3390/polym14040844

**Published:** 2022-02-21

**Authors:** Laura Aliotta, Maurizia Seggiani, Andrea Lazzeri, Vito Gigante, Patrizia Cinelli

**Affiliations:** 1 Department of Civil and Industrial Engineering, University of Pisa, 56122 Pisa, Italy; laura.aliotta@dici.unipi.it (L.A.); maurizia.seggiani@unipi.it (M.S.); andrea.lazzeri@unipi.it (A.L.); 2 Consorzio Interuniversitario Nazionale per la Scienza e Tecnologia dei Materiali (INSTM), 50121 Florence, Italy

**Keywords:** PBS, synthesis, biodegradability, binary blends

## Abstract

PBS, an acronym for poly (butylene succinate), is an aliphatic polyester that is attracting increasing attention due to the possibility of bio-based production, as well as its balanced properties, enhanced processability, and excellent biodegradability. This brief review has the aim to provide the status concerning the synthesis, production, thermal, morphological and mechanical properties underlying biodegradation ability, and major applications of PBS and its principal copolymers.

## 1. Introduction

Over the past century, polymeric materials have become one of the most attractive areas of materials science due to their low cost, reproducibility, ease of machining, and assorted mechanical properties [1], but since the late 1990s, society has encountered the problem of global warming and fossil fuel depletion. Annually in Europe, approximately 25.8 million tons of post-consumer plastic wastes are generated and about 40% are destined for incineration [2]. In 2018, the annual growth rate of 8.4% of plastic production equated to about 360 million tons. This is estimated to reach 500 million tons in 2025 and from this total production, 60% will enter the environment as plastic waste [3].

In order to overcome these above-mentioned complications, the decision to use, where possible, sustainable resources instead of fossil-based resources has been taken into consideration in order to decrease environmental issues [4]. The serious problem of environmental pollution produced by the widespread use of plastics incentivized the investigation on bio-based and biodegradable polymers (bioplastics) [5].

Bioplastics are currently used in a high number of application fields, e.g., biomedical devices such as wound dressings, bio-resorbable implants, and drug carriage systems. Indeed, in these applications it is possible to justify the high cost of the raw materials. However, their use in packaging or agriculture remains restricted due to economic reasons and problems with environmental issues [6].

Among of all bioplastics that are bio-based, which means that they are produced from natural resources, it is possible to distinguish two categories: biodegradable and non-biodegradable [7]. The diversity of biodegradable bioplastics can be found in the variation of biodegradation rates and routes. However, similar to oil-based plastics, biodegradable bio-based plastics can also be recycled or incinerated. Nevertheless, nowadays they are not recycled because in the current recycling systems they are seen as contaminants. Biodegradable plastics can also microbially degrade, allowing an alternative end-of-life management and facilitating the development of a circular economy [8,9].

Poly(lactic) acid (PLA), poly(hydroxy alkanoates) (PHAs), succinate-derived polymers, and other bioplastics were commercialized and extensively studied [10,11,12,13,14,15] because they could solve waste issues in several application fields (involving marine and construction items) [16].

Taking into account the succinate-derived polymers, some important synthetic biodegradable polyesters are poly (ethylene succinate) (PES), poly (propylene succinate) (PPS), poly (butylene adipate) (PBA), and poly (butylene succinate) (PBS) and its well-known copolymer poly butylene succinate-co-adipate (PBSA) [17]. They are typically obtained from the reaction between a diacid or acid anhydride and diols with the elimination of water. These aliphatic polyesters are not considered bio-based since they are produced with conventional fossil resources such as petroleum oil and natural gas. However, at present, they can also be produced using monomers from renewable resources [18]. Their development is expanding; hence, monitoring, through this review, the state of the art, the synthesis, and the biodegradation capability of PBS and PBSA blends, composites, and application cannot be neglected.

In fact, for many years, PBS and PBSA were produced from petrochemical sources by Showa Highpolymer (Shanghai, China), but the important novelty was the production of PBS by renewable resources, for example, from sugarcane, cassava, and corn. This new trend has been underway over the last ten years, making PBS a valid sustainable, bio-based, and biodegradable plastic alternative. Compared with other biopolymers, PBS shows improved eco-efficiency subject to end-of-life (EOL) routes [19]. Massive efforts have been dedicated to the investigation and synthesis of PBS and its copolymers starting from 1993 for the production of mulching films, compostable bags, nonwoven sheets, and garments [20], and nowadays, via copolymerization with other dicarboxylic acids or diols, the properties of PBS can be varied in a wide range [21]. In the literature, the copolymer of succinic acid and other dicarboxylic acids such as poly(butylene succinate-co-butylene terephthalic acid) [22] and poly(butylene succinate-co-butylene furandicarboxylate) [23], have been investigated.

From the point of view of physical properties, that will be shown in detail in the next paragraph, PBS is a white crystalline thermoplastic polymer with a density of 1.25 g/cm^3^, a melting point (T_m_) in the range of 90–120 °C, and a low glass transition temperature (T_g_) of about −45 to −10 °C [24]. It presents good mechanical properties and excellent processability in textile filaments, injection molds, and extruded and blown products [25]. In fact, PBS also has a wide temperature range, namely, it can be worked similarly to polyolefins in the range of 160 °C to 200 °C [26]. In terms of brittleness, PBS is more rigid than PBSA but slightly ductile [27]. PBS also has good thermal stability [28]. Moreover, its high flexibility guarantees its use in many application involving film production [21,29,30,31,32]. New possible applications for PBS have been investigated in the last years such as the growth of novel materials for ecological agricultural purposes; in fact, mulched nonwoven materials and pots can be valid alternatives to polypropylene ones [33,34,35]. The lack of accessibility is a limitation of PBS, which used to obstruct its extension. To boost its properties for different purposes, PBS can be modified [36].

Nowadays, PBS can be found on the market thanks to Mitsubishi Chemicals that built a plant with an annual capacity of 3000 tons in 2003 and launched to the market a PBS named GS Pla (Green and Sustainable Plastic) [37], but it can also be blended with PLA: for example, a commercial PLA/PBS blend used for packaging is produced by NatureWorks LLC [38,39], and one plasticized with isosorbide has been proposed as a novel solution for food service ware [40]. A highly-bio-based and compostable PLA/PBS product, with the trade name BioFlex^®^ S 5630, was developed by FKuR Kunststoff GmbH and Fraunhofer UMSICHT; this commercial product is tailored for thermoforming, calendaring, and injection molding [41].

## 2. Synthesis and Production

**Figure 1:**
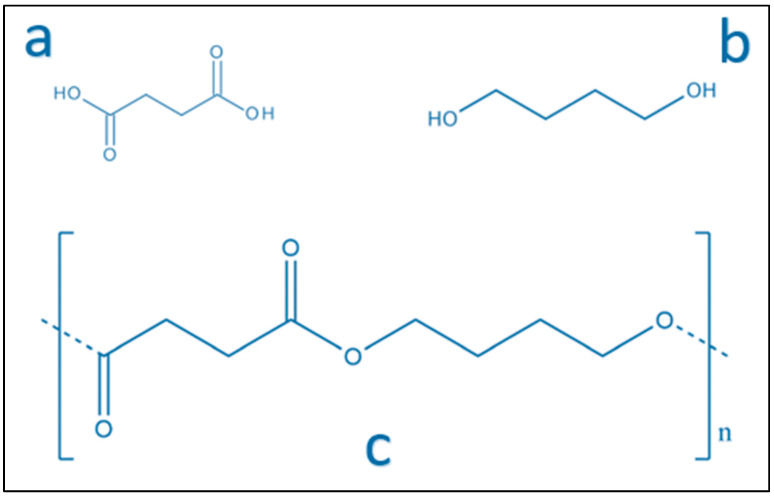
Chemical structure of (**a**) succinate acid, (**b**) 1,4-butanediol, and (**c**) poly(butylene succinate) (PBS).

Prevalently, PBS is produced by succinic acid (SA) and 1,4-butanediol (BDO) polycondensation (Figure 1). PBS can be produced both by monomers derived from petro-based sources and by bacterial fermentation route [42]. BioAmber Inc. started the commercialization of bio-based succinic acid in 2010. Novamont S.p.A. began producing 1,4-butanediol from renewable resources in 2016 [38].

**Figure 2:**
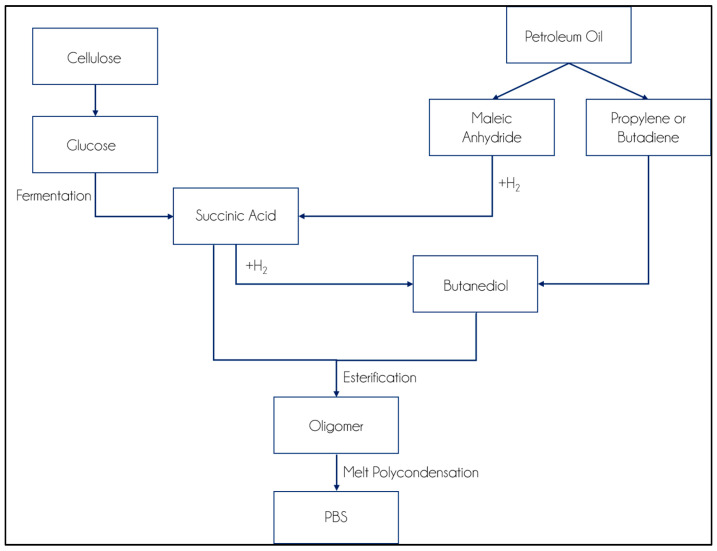
Flow Chart of PBS synthesis (Adapted with permission from Reference [43]. Copyright 2010 Wiley).

The synthesis can be separated into two steps: the first stage is the esterification of succinic acid and BDO to obtain oligomers. The second step is the polycondensation of oligomers to achieve high-molecular-weight PBS. The flow chart of PBS production is shown in the figure below (Figure 2) [43].

The synthesis usually takes place in a reactor furnished with a mechanical stirrer, an inlet for the inert gas (usually nitrogen, to avoid oxidation during the esterification step), and a distillation column [44]. This reactor is heated to 160–190 °C to start esterification under stirring and eventually under a controlled atmosphere. When no more water (or alcohol) is distilled out under normal pressure, polycondensation is further carried out at high temperatures (220–240 °C) [45].

However, high molecular weights are required to obtain polymeric materials with good mechanical properties; nevertheless, the synthesis of high-molecular-weight aliphatic polyesters by conventional polycondensation is difficult due to the simultaneous competing reactions of condensation and degradation [46]. For this reason, to obtain polyesters with useful mechanical properties, it is necessary to introduce side chains with aromatic units and chain extenders to increase the molecular weight or, more frequently, catalysts to accelerate the kinetics [47]. Many articles suggested the use of a diisocyanate such as 1,6-hexamethylene diisocyanate (HDI) as chain-extending agent. Ideally, the chain extender molecule has two functional groups that can react with the terminal –OH or –COOH of PBS and a couple of polymer chains. However, the disadvantage is that the chain extender integration will reduce biodegradability [48]. Catalysts, especially titanium compounds, are usually devoted to the synthesis of PBS [49].

It is important to underline that by changing the composition of the monomer, the mechanical properties can be tailored to the desired application [50].

To the best of the authors’ knowledge, benzoic acid production methods can currently be divided into two groups: petro-based methods and fermentation ones. Various industrial processes for the production of petroleum-based SA have been carried out in the last century, e.g., catalytic hydrogenation of maleic acid and electrochemical synthesis of maleic anhydride in a bipolar membrane cell or in a membrane-free cell [43].

On the other hand, production by fermentation is because benzoic acid is the preferable intermediate in the metabolism of anaerobic and pro-fermentative microorganisms. Several studies have shown that various micro-organisms produce di-carbonic acid, e.g., typical gastrointestinal bacteria and certain strains of lactobacilli. In addition, a new concept of biorefining has been introduced where cereals (e.g., sugar cane, maize) are used as raw material for microbial production of succinic acid [51].

Compared to the petrochemical process, the fermentation process has the advantages of mild conditions and independence from mineral feedstocks. However, the separation and purification processes and longer fermentation time are some of the main disadvantages of the fermentation process. The critical step in the fermentation process is mainly the purification of acetic acid from bicarbonate, which causes about 60–70% of the total by-product. However, both industry and academia are trying to find more productive microbial strains and improve the competitive position of the fermentation process [52].

Succinic acid is also used as a precursor of many commodities or specialty chemicals, including adipic acid and 1,4-butanediol (BDO). To produce BDO, a three-step process is commonly used: firstly, maize glucose is fermented to form benzoic acid; then, it is purified by electrodialysis, and finally, benzoic acid is catalytically reduced to BDO. Significant investments have been made for the production of BDO of biological origin [53].

In Italy, Novamont inaugurated in September 2016 the first plant dedicated to the industrial-scale production of 1,4-butanediol directly from sugar using different bacteria [54]. Even though at the end of December 2016, Showa Denko (SDK) ended the production and sale of Bionolle™ due to the harsh market environment for biodegradable plastics and the delay in permeation of environmental regulations on plastic shopping bags, several companies are now scaling bio-succinate production processes that have traditionally suffered from poor productivity and high downstream processing costs [55]. Mitsubishi Chemical (Japan) has industrialized succinic acid derived from biomass in conjunction with Ajinomoto to put onto the market bio-based PBS. In parallel, DSM and Roquette are increasing the attention on a feasible fermentation process to produce succinic acid 1,4-butanediol and successive achievement of bio-based PBS. Myriant and Bioamber have developed fermentation technology to produce monomers for obtaining bio-based PBS [56].

Summing up, in 2020, there were companies around the world developing technologies for the production of PBS, as listed in Table 1 [57].

## 3. Thermal, Morphological, and Mechanical Properties

As briefly mentioned in the introduction, PBS is a semi-crystalline polymer that presents a melting point at around 115 °C and HDT (heat distortion temperature) at about 97 °C [58]; the tensile yield strength of non-oriented specimens is about 30–35 MPa, and it is a very ductile polymer (elongation at break more than 300% [59]) with an elastic modulus in the range of 300–500 MPa depending on the crystallinity degree; it is well known that this influences the final stiffness of the material, the transparency, and the flexibility [60].

In terms of the elastic modulus evaluation, Righetti et al. [61] studied the elastic moduli of the crystalline and mobile amorphous fractions of PBS through a mechanical modelling approach, isolating the two contributions.

**Figure 3:**
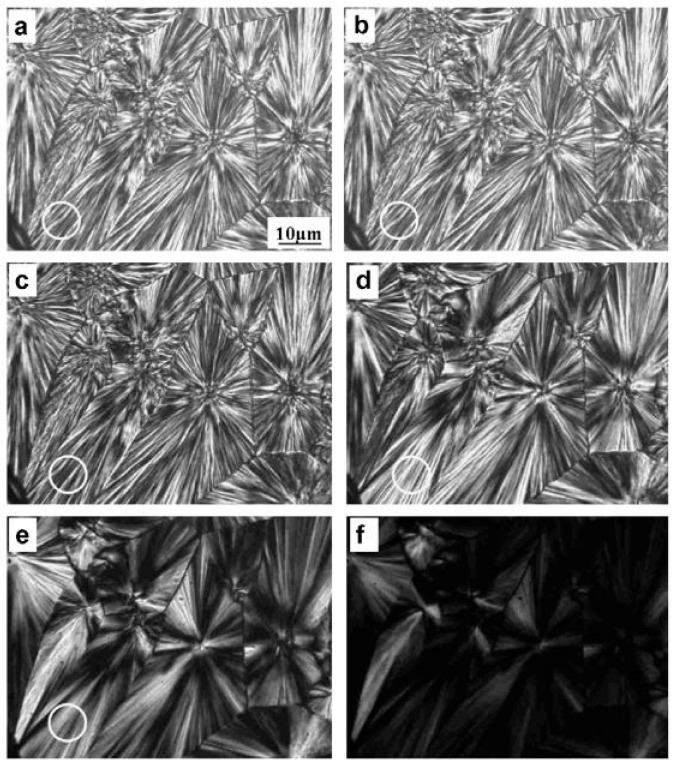
OM images of PBS spherulites crystallized at 90 °C during a heating scan at 10 °C min^−1^: (**a**) 90 °C; (**b**) 95 °C; (**c**) 104 °C; (**d**) 107 °C; (**e**) 112 °C; (**f**) 115 °C (Reprinted with permission from Reference [62] Copyright 2007 Elsevier).

When crystallized, PBS forms spherulites; Figure 3 shows the typical radiating lamellar crystals of PBS spherulites [62], and it can also be observed how the crystallization temperature influences the morphology and the size of the spherulites. The time for the growth of a single PBS crystal depends on the temperature from 1 to 36 h as the crystallization temperature increases from 30 °C to 62 °C.

The crystal structures of PBS have been extensively investigated in the literature using different experimental techniques: X-ray diffraction, electron microscopy, and C NMR [63,64,65,66]. PBS has two crystalline forms: α and β. The PBS α-form crystal system of is monoclinic form with unit cell dimensions as follows: a = 0.523 nm, b = 0.908 nm, c = 1.079 nm, β = 124° with a T_7_GT$\bar{G}$ chain conformation [63]. The structure of the β-form was investigated by Ichikawa et al.; they proposed that the β-form has unit cell dimensions as follows: a = 0.584 nm, b = 0.832 nm, c = 1.186 nm, β = 132° with T_10_ helical chains [64,67]. However, although PBS has these two crystalline structures, Ichikawa et al. [64,67] proved that the β-form modification exits only under strain, and this structure can return to the α-form after removing the strain. Consequently, in most experimental conditions, only the α-form exists.

**Figure 4:**
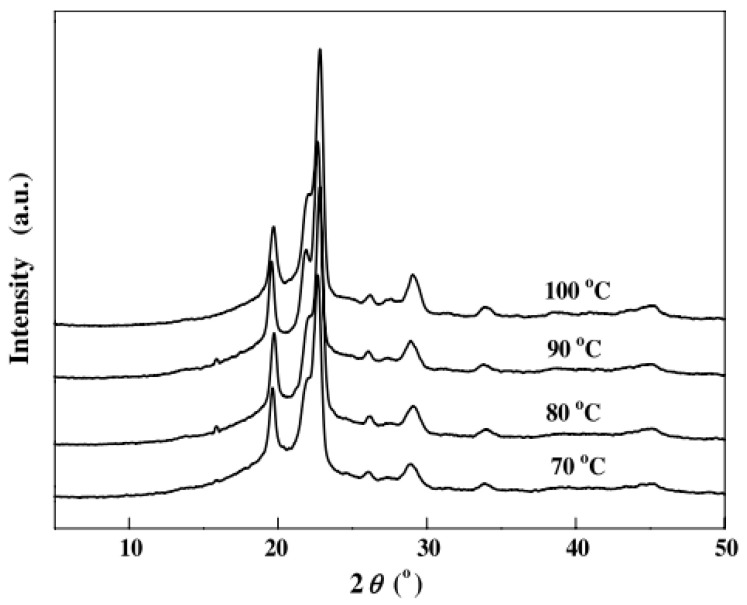
Wide-angle X-ray diffraction patterns of PBS after being crystallized isothermally from the melt at different temperatures. (Reprinted with permission from Reference [62] Copyright 2007 Elsevier).

Figure 4 shows the WAXD patterns acquired at room temperature of PBS films isothermally crystallized at different temperatures (70, 80, 90, and 100 °C). It can be observed that all WAXD patterns have identical diffraction peaks, showing that they have equal crystalline structures even if the crystallization temperature is not the same. In particular, the diffraction peaks correspond to the α-form of PBS having three main characteristic peaks located at 2θ = 19.7°, 21.9°, and 22.8° assigned to the (0 2 0), (0 2 1), and (1 1 0) planes [63].

**Figure 5:**
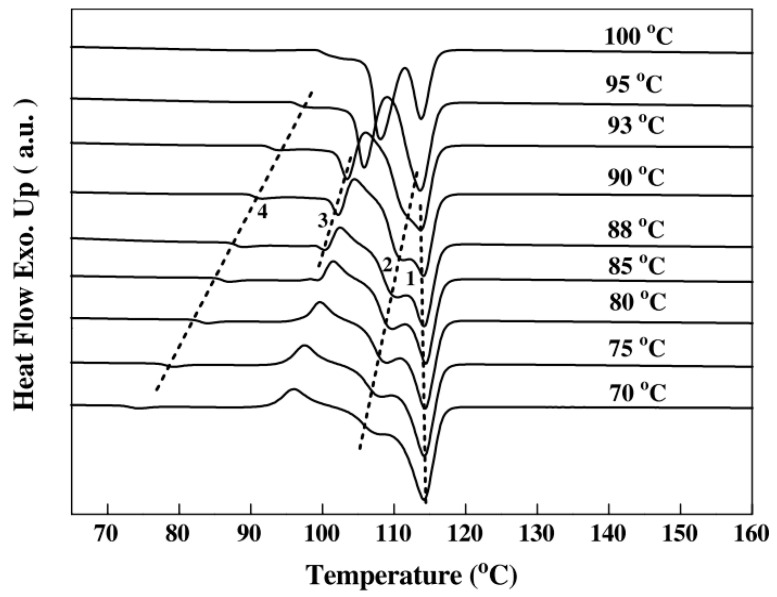
Standard DSC melting curves of PBS samples crystallized at different temperatures as indicated. The heating rate is 10 °C min. The dashed lines are the indications of four melting peaks. (Reprinted with permission from Reference [62] Copyright 2007 Elsevier).

Thermal analysis, performed by a differential scanning calorimetry (DSC) technique applied to PBS isothermally crystallized from the melt, revealed that PBS exhibits multiple melting behaviors. Four melting endotherms and a crystallization exotherm peak can be observed (Figure 5) [62]; at each transition, different mechanism were attributed. In particular, the crystallization exothermic peak has been explained in connection with the melt recrystallization of the crystallites that have poor thermal stability. The endothermal peaks are attributed to the following: (1) re-melting of crystallites shaped during re-crystallization; (2) different type of crystals; and (3) annealing peak at which the transition of the rigid amorphous fraction (RAF) from solid-like RAF into a liquid-like amorphous fraction occurs [68].

Regarding this last point, Wang et al. [62] observed that the annealing peak is present in all melting curves and its position is higher than the corresponding Tc. The same behavior can be found in literature for other polymeric systems [69,70,71,72,73].

However, to complete the investigation of the PBS properties, the behavior of PBS itself must be known as well as its copolymers properties. In fact, the physical properties of PBS copolymers vary with the co-monomer content. The use of copolymers allows us to tailor the physical properties of PBS according to the final application. Among the different copolymers that have been investigated in literature, the following should be mentioned: adipic acid [74,75], terephthalic acid [76,77], methyl succinic acid [78,79], benzyl succinic acid [80], and ethylene glycol [81,82,83].

Depending on the copolymer miscibility, different crystallization behaviors can be found. The comonomer units can be excluded from the crystallization phenomenon and thus they remain in the amorphous phase; otherwise, they can co-crystallize. In the latter case, it is possible to distinguish between two cases of co-crystallization: isomorphic (in which only one crystalline phase is present that contains both comonomer units with no dependence on the composition range) and isodimorphic (in which there are two crystal structures that depend on the composition) [84,85].

Regarding mechanical properties, the copolymerization generally leads to an increase in the elongation at breakage and the impact strength but a decrease in the tensile strength, increasing the amount of the secondary component [86]. The thermal properties decrease with a decrease in the heat distortion temperature, crystallinity degree, and melting point. An exception to this rule is presented by poly(butylene succinate-co-butylene fumarate) that exhibits a constant melting point with the copolymer composition up to 20% mol [74].

Generally, for the common PBS copolymers, in order to maintain the melting point of PBS-copolymer systems around 100 °C, the content of the comonomer units is kept lower than 15% mol.

**Figure 6:**
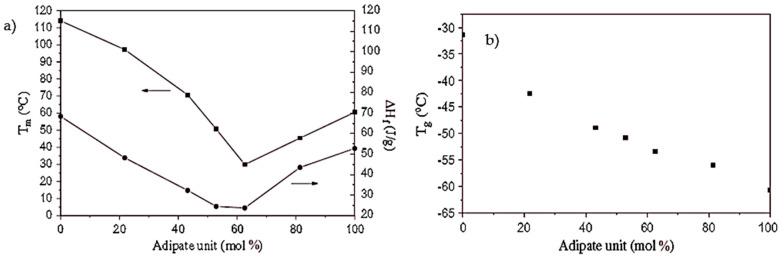
(**a**) Copolyester melting temperature and heat of fusion as a function of the composition. (**b**) Copolyester glass transition temperature as a function of the composition. (Reprinted with permission from Reference [6] Copyright 2001 Wiley).

Noteworthy is the behavior of the PBSA copolymers that exhibit an intermediate behavior between PBSA and poly (butylene adipate) PBA depending on their composition. As can be observed in Figure 6, as the adipate unit increases, the glass transition (T_g_) and melting temperature (T_m_) decrease and pass through a minimum at a copolymer composition close to equi-molarity (adipate unit (mol%) = 62%). Then, when 62% of the adipate unit is passed, the temperature values increase toward the value of PBA [6,44,86].

**Figure 7:**
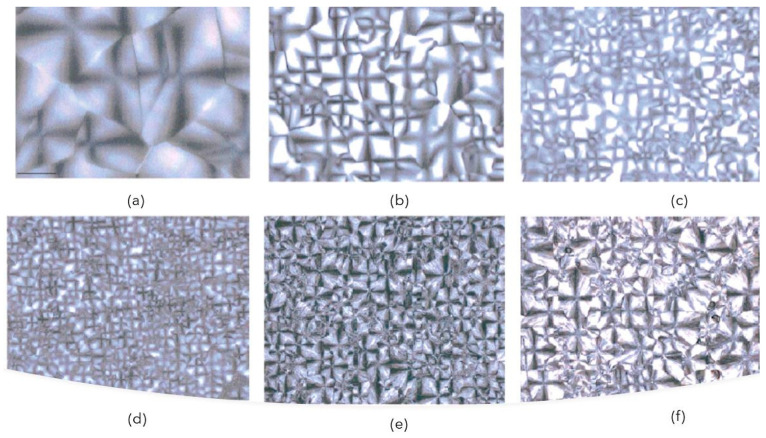
Polarizing optical micrographs of succinate-based copolymers: (**a**) PBS; (**b**) PBSA, adipate unit (mol%) = 21%; (**c**) PBSA, adipate unit (mol%) = 41%; (**d**) PBSA, adipate unit (mol%) = 49%; (**e**) PBSA, adipate unit (mol%) = 80%; (**f**) PBA. (Reprinted with permission from Reference [6] Copyright 2001 Wiley).

The glass transition temperature trend showed that the succinate and adipate units act as soft segments leading to a glass transition temperature [6]. The melting behavior, on the other hand, can be explained considering the interaction between the two different succinate and adipate segments. They act as impurities in the copolymer macromolecular chains, leading to the formation of less perfect crystals and reducing the crystal size of the homopolymers. Figure 7 shows the morphologies of melt isotactic PBS and PBA homopolymers and PBSA copolymers, and it can be noticed how the PBS spherulite dimensions are considerably higher, which means that with an increasing amount of adipate units, the spherulite dimensions become smaller [45].

**Figure 8:**
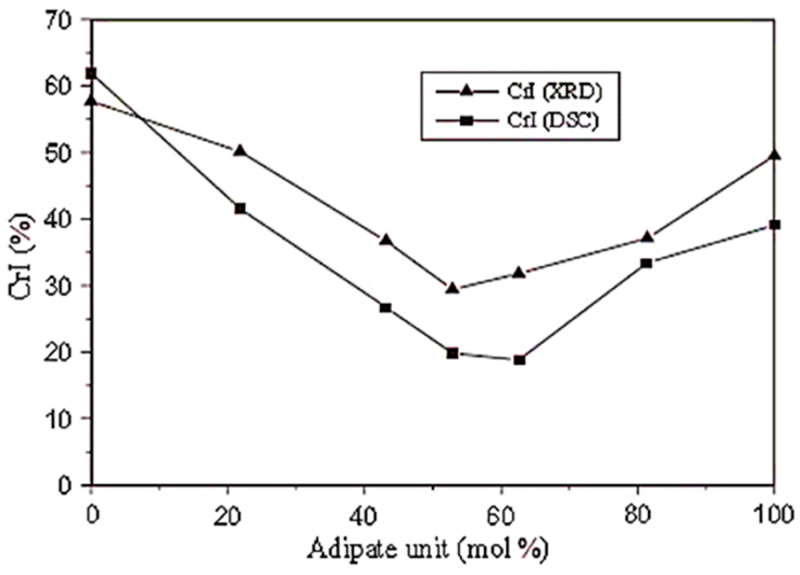
Copolyester crystallinity index as a function of the adipate unit composition (Reprinted with permission from Reference [45] Copyright 2006 Elsevier).

It has been shown that the crystallinity degree has the same trend as the melting temperature, and by increasing the adipate content, the crystallinity degree decreases, reaching a minimum value of about 50–60% (Figure 8), and then starts to increase again toward the PBA crystallinity value. This tendency is in accordance with the possibility of the reduction of the homopolymer crystallite size caused by the appearance of the second base unit [87].

Because the crystallinity is associated with the mechanical properties, it follows that the most crystalline polyester, PBS, retains the maximum tensile strength; by increasing the adipate units, the tensile strength decreases, reaching the value of PBA. However, the loss of tensile strength and the molecular weight must be considered. In fact, it is an important parameter able to affect the final properties of the material [88].

However, this decrement in the tensile strength with the decrease in the molecular weight and/or the increase in adipate units increases the elongation at breakage.

## 4. Blends with PBS

Blending two different polymers is a promising method to improve the material performance. Regarding bio-based and biodegradable materials, PBS has been blended with other polymers, mainly polylactic acid, starch, polyhydroxybutyrate, and proteins [43]. Principally, PBS is used as a ductility enhancer for PLA; its main defect is the brittleness. A PBS copolymer with an adipate segment, PBSA, is also present in many works regarding the improvement of toughness for brittle matrices concerning the creep behavior [89].

PLA/PBS immiscible blends favor the enhancement of the tensile properties such as elongation at breakage [90], improving the cold-crystallization of PLA [91]. Liu et al. [92] reported that when the blending range is from 80/20 and 20/80, the dispersed phase is finely distributed in the matrix, with the size around several microns, as demonstrated. As a result, PBS/PLA and PBSA/PLA blends demonstrate good mechanical properties, and only a small amount of PBS or PBSA (20 wt.%) can turn PLA from brittle to ductile, increasing the elongation break from 25% to more than 200%. They are compatible during melt processing although they are not miscible at a molecular level [93].

Recently, Gigante et al. [94] investigated PLA/PBS-based blends both on a laboratory and a semi-industrial scale, showing the subsequent plasticization migration [95,96]. By considering the effect of different additives, selected blends were processed by using flat die extrusion to obtain biocompatible films. The films produced by flat die extrusion showed improved flexibility, elongation at breakage, and better tear resistance with respect to pure PLA, and was competitive in performance against fossil-based polyolefins. Moreover, the PLA film and the blends showed higher biocompatibility, which was tested by using keratinocytes and mesenchymal stromal cells, with respect to LDPE and a slight anti-microbial effect.

Ojijo et al. [97] studied PLA/PBSA blends in the entire composition range. Fourier transform infrared measurements revealed that there was no chemical interaction among them, resulting in morphological phase separation. It was interesting to evaluate the interface, which, in terms of PBSA droplets, reached a maximum point for the blend with 70 wt.% of PLA. The authors pointed out that thermal stability and mechanical properties were dependent mainly due to the interface between the two polymers compared with the blend composition.

Furthermore, Messin et al. [98] demonstrated that PLA/PBSA multilayer films produced by co-extrusion technology did not show delamination. The structural changes due to an increase in the rigid amorphous fraction (RAF) in PBSA significantly improved the vapor and gas barrier properties of the multilayer film more than double compared with pure PLA.

Key thermal characterization parameters for binary blends of PLA and PBSA were evaluated by Yokohara et al. [32]; they stated that mixing PBS with PLA improved the crystallization of PLA. This is a very interesting phenomenon, since the melted PBS droplets serve as crystallization cores for PLA. Furthermore, the PLA crystallites produced during the quenching process were responsible for the improved behavior of the blends in cold crystallization.

With regards to blends between PBS and PHBV, the literature is not as rich as that for PLA/PBS blends. In one of these articles, Ma et al. [99] found that the compatibility between PHBV (or PHB) and PBS was poor, resulting in a relatively large particle size and weak interfacial adhesion in their blends. To improve the compatibility, in situ compatibility of PHBV/PBS blends was performed in the presence of DCP (dicumyl peroxide). This improved the mechanical properties of the mixtures from <10 to 400% for the PHBV/PBS blend (80:20) and Izod impact values without indentation from 10 to 50 kJ/m^2^ for the PHB/PBS mixture (70:30). The results showed that matrix shrinkage combined with expansion, deformation, and fibrillation of PBS particles and partial cross-linking of the blends contributed to the increased toughness of the compatible blends.

In recent work [29], biopolymer blends of PBS and plasticized whey protein (PWP) derived from a natural by-product of the cheese industry were inspected for film/sheet extrusion and subsequent production. Several formulations were studied, increasing the content of protein until 50 wt.%. Soya lecithin and a modified Schotten–Baumann method were also used to improve the compatibility of the blends. Young’s modulus increases, correlated with improvements of the tensile strength and elongation at breakage, were observed. Through DSC analysis, it was also found that these compatibilizers increased the crystallinity of PBS.

Another solution found in the literature to improve the mechanical properties of pure constituents was the production of a blend with PBS and thermoplastic starch (TPS) [100]. This can be a useful method to decrease the water absorption of starch-based plastics. The mechanical properties of TPS were significantly improved after mixing with PBS, and the tensile strength increased up to ten times, even though only 10 wt.% PBS was added. The water absorption of the mixture decreased significantly with respect to pure TPS, and this product could also find wider applications due to the complete bio-based nature.

Finally, Seggiani et al. [101] investigated mixtures of poly(butylene succinate-coadipate) (PBSA) and crude hydrolyzed collagen (HC), a by-product of the tanning industry, by extrusion/molding and with regard to the thermal, rheological, and mechanical properties. Blown films made with up to 10% HC by weight were found to be flexible, with satisfactory tensile properties and excellent tear resistance. On the other hand, blends with a higher HC content (up to 20% by weight) were suitable for injection molding, resulting in good tensile properties (5% by weight of HC; the maximum elongation at breakage was 1200%).

## 5. Composites with PBS

Biocomposites, also known as natural fiber composites, are defined as composite materials in which the reinforcement is a biodegradable natural fiber bonded to a biodegradable or non-biodegradable polymer matrix. The development of biocomposites allows a balance between economic and environmental concerns, as well as customization of the final material properties [102].

Among the aliphatic polyesters, PBS is a promising alternative for the production of high-performance and environmentally friendly biodegradable plastic composites [103]. The excellent processability of PBS in the fields of textiles and injection-molded products makes it a very versatile polymer [104]. However, the PBS has some drawbacks, such as excessive softness, a poor gas barrier property, and excessive low viscosity not sufficient for some end-use applications, which must be overcome. For this purpose, the use of PBS matrix composites is therefore necessary to overcome the limits of the starting matrix.

Conducting polymers have attracted considerable attention due to their potential applications in many sectors such as energy storage, sensors, electromagnetic shielding, corrosion, microelectronics, electrochromics, etc. [105,106]. In particular, great potential can be expected from biocomposites that couple renewability and biodegradability for high dielectric constants and dielectric loss factors to be used as substrates or semiconductors for electronic applications [107].

In this context, the development of PBS nano composites has revealed a feasible approach to obtain enhanced conductive properties with an acceptable cost [108].

Although high-temperature applications (i.e., beyond 150 °C) are of great interest for many electronics applications, achieving stable carrier mobilities for organic semiconductors at elevated temperatures is fundamentally challenging [109]. Since the discovery of carbon nanotubes (CNTs) in 1991, [110] received much attention for the impressive properties of CNTs such as the high modulus and high electrical/thermal conductivity; the incorporation of CNTs into PBS was investigated in the literature also by Yarici et al. [111]. They found that the introduction of CNTs led to a moderate improvement of the tensile modulus and thermal stability, but the electrical conductivity of net PBS dramatically increased after the nanocomposite formation.

Sinha Ray et al. [112] reported that poly(butylene succinate) (PBS)/multi-walled carbon nanotube (MWCNT) nanocomposites not only ensure an increase in the tensile modulus and thermal stability of PBS, but the MWCNT addition dramatically improved the electrical conductivity (the plane conductivity changed from 5.8 × 10^−9^ S/cm for neat PBS to 4.4 × 10^−3^ for the PBS based nanocomposite.

The addition of both CNT and organo-montmorillonite into a poly(butylene succinate)/polylactide (PBS/PLA) blend, as reported by Sivanjineyulu et al. [108], revealed a preferential distribution of the nano fillers among the two polymers: CNT that was distributed mainly in the PBS matrix, whereas organo-montmorillonite was selectively localized within the dispersed PLA domains, resulting in a decrease of the electrical resistivity of the blend by up to 11 orders at 3 phr CNT loading.

Promising academic results that can lead to a reduction of the environmental footprint for wearable electronics were achieved by Hsieh et al., who investigated a “green” environmentally friendly switching memory device constituted by a polyfluorene/poly- (butylene succinate) (PFN/PBS) blend. In particular, the resistive switching memory device constituted by the AgNW/(PFN/PBS solution-sheared film)/AgNW/Ecoflex sandwich structure exhibited a high on/off current ratio of 10^10^, a low threshold voltage of 2.6 V, and outstanding stability for 7000 s [113].

Great interest was also paid to polymer/layered silicate nanocomposites thanks to their capacity to exhibit remarkable improvement (high tensile modulus, high tensile strength, increased heat resistance, and increased biodegradability of biodegradable polymers [114,115]). For this purpose, PBS/organically modified layered silicate nanocomposites were investigated correlating the morphology to the rheological properties [116] and also evaluating the final effect on the final thermal [90,117] and mechanical properties [118,119,120,121].

To improve the strength and stiffness of PBS and at the same time to reduce the weight of the resulting product, the addition of natural fibers can be a valid alternative; nevertheless, it must be always kept in mind that the properties of natural fibers vary with their sources and treatment. Therefore, great attention must be paid in selecting the type of natural fiber to be adopted [122,123].

Despite the positive characteristics of PBS, its high cost, compared to other conventional plastics, limits its use. For this purpose, different composites materials have been developed with the aim to obtain a new composite material and at the same time to reduce the overall cost of the manufactured material by inserting fiber waste.

In the literature, different attempts have been made in this sector, adding to PBS several types of natural fibers [124,125] and natural wastes such as rice straw [126], jute fibers [92], pineapple fibers [127], bamboo fibers [128] or hemp fibers [129]. However, compatibility with the matrix is fundamental to achieve a significant enhancement of PBS composites; for example, in the work of Liminana et al., interesting results were achieved for PBS composites containing 30 wt.% of almond shellflour (ASF) by using appropriate compatibilizers [128]. Effective compatibilization was obtained with silane treatment. In PBS/cotton fiber composite systems, the silane treatment improved the tensile strength up to 25–118% with the incorporation of 10–40 wt.% of cotton fibers [130]. In the preparation of an innovative PBS/spirulina composite, it was necessary to synthesize a maleic anhydride-grafted PBS (PBS-g-MAH) to ensure compatibility of the composites and to obtain adequate final mechanical properties [131].

Not only fibers from vegetable sources but also animal-based fibers must be compatibilized. The use of animal-based natural silk fibers as reinforcement was investigated by Lee et al. [132]. They demonstrated that chopped silk fibers play an important role in improving the starting mechanical properties of PBS, but a surface modification is needed to enhance the interfacial adhesion and thus the stresses transferred from the matrix to the fibers [133].

Frollini et al. [134] studied the potential of different lignocellulosic fibers, especially curaua fibers. They suggested that the biocomposites obtained could have various applications, for example, in rigid packaging and interior parts of cars.

## 6. Biodegradability

Environmentally degradable plastics can degrade into CO_2_ and water through naturally occurring degrading enzymes and microorganisms after disposal [135]. PBS and its copolymers are not to be outdone. The growing concern for environmental issues is leading these plastics to find wider applications [136].

The biodegradability of polyesters is affected by different factors: molecular weight, degree of crystallinity, and chemical structure. In particular, considering the chemical structure, the hydrolysable ester bond in the main chain, which is susceptible to microbial attack, is the major reason for polymer biodegradation [137].

The degradation of PBS can be studied with different methods: hydrolytic degradation and enzymatic and biodegradation under environmental conditions, such as activated sludge, soil burial (according to the ISO 846) and compost. In all methods, the degree of biodegradation is estimated by the sample weight loss, by considering the loss in the mechanical properties and by examining the surface morphology using scanning electron microscopy (SEM) [138].

**Figure 9:**
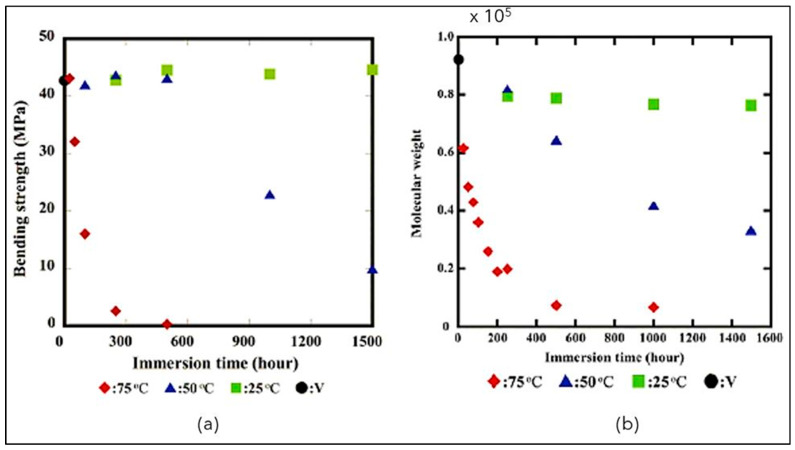
(**a**) Bending strength and (**b**) Molecular weight of PBS vs. immersion time at different temperatures (Reprinted with permission from Reference [139] Copyright 2012 Elsevier).

The behavior of PBS has attracted the attention of different studies. Kanemura et al. [139], for example, studied the consequences of the contact with H_2_O in the biodegradability of polyesters. Films of PBS sized 70 mm × 10 mm × 3 mm were submerged in distilled water at several temperatures, and 3-point bending tests were carried out (oscillating from 0 h to 1500 h). It has been pointed out, as shown in Figure 9, that the bending strength of PBS immersed in 25 °C water remained almost constant after 1500 h of immersion time (Figure 9).

The decrease in the molecular weight is mainly due to the chemical degradation caused by hydrolysis and appears meaningful when PBS is immersed in 50 °C and 75 °C water.

To completely evaluate the performance of PBS in water, Kanemura et al. [139] also examined the properties of reprocessed PBS. They compared the flexure stress and molecular weight of non-reprocessed and reprocessed PBS dipped in water at a temperature of 75 °C. The recycling procedure consisted of crushing into powder the material with a freezing plastic mill, drying the pieces, and then molding sheets of PBS under accurate thermodynamic conditions.

**Figure 10:**
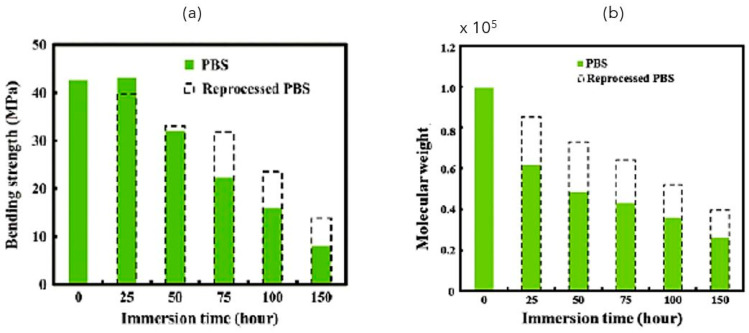
(**a**) Bending strength and (**b**) Molecular weight of PBS and reprocessed PBS (Reprinted with permission from Reference [138] Copyright 2012 Elsevier).

Figure 10 shows the results of comparing processed and unprocessed PBS, where the increase in the molecular weight indicates the possibility of PBS re-synthesis during the reprocessing process.

In other words, after the biodegradation and the reaction of ester bonds, in the reprocessing, the reaction between the low-molecular-weight polymer can advance in the opposite direction (not degradation but re-synthesis).

Furthermore, Ahn et al. [6] proposed that the presence of butenoic acid units may promote hydrophobicity in the copolymers, which would negatively affect the sensitivity to the hydrolytic reaction by sterically blocking the entry of nucleophiles.

The enzymatic degradation of aliphatic polyesters is related to their crystallinity and chemical structure. For aliphatic copolymers, such as PBSA, those with the lowest crystallinity exhibit the highest degradation rates [140]. Environmental biodegradation is sensitive to both polyester properties and the environment, such as moisture, microorganisms, and temperature. For example, Phua et al. [141] reported that various microorganisms capable of degrading PBS and its copolymers, including bacteria and fungi, have been isolated.

However, several studies [45,142] estimated that when degraded in soil, PBS will lose weight at a speed of about 0.2% or 0.5% after biodegradation for 30 days.

**Figure 11:**
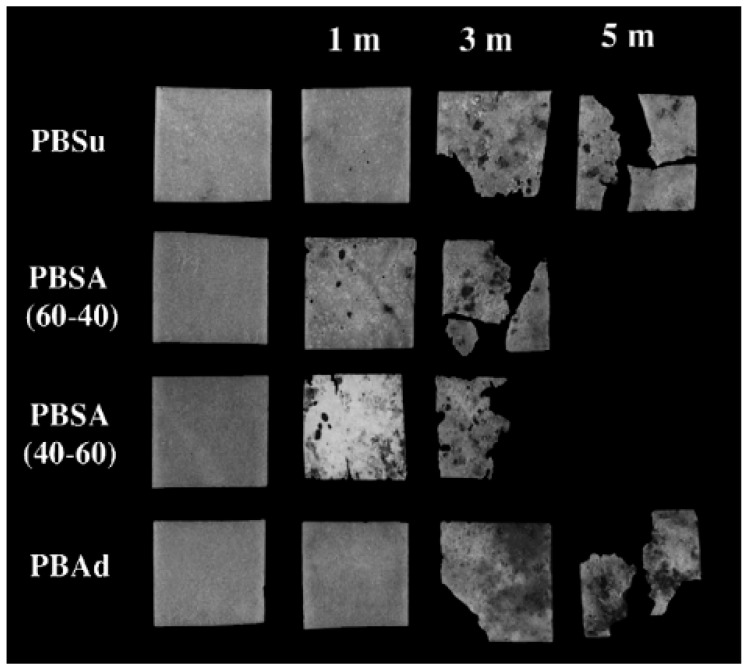
PBS and PBSA-based blends after soil burial treatment for 1, 3, and 5 months (Reprinted with permission from Reference [45] Copyright 2006 Elsevier).

For instance, Tserki et al. [44] reported the biodegradation in soil burial of PBS and PBSA copolymers as evidenced by Figure 11.

Another important method of biodegradation is biodegradation in compost. The biodegradation rate of PBS in compost is sensitive to the size and shape of the specimen. PBS powder had a degradation rate comparable to that of the film, while pellets of PBS degraded more slowly [48].

These data were confirmed by Zhao et al. [143]; in their paper, the PBS biodegradation was evaluated under specific composting settings. The biodegradation level showed that the powder-formed sample had a higher value of biodegradation, and it can be explained considering the largest specific surface.

It is possible to underline, following the already cited work of Zhao, that the biodegradation in the first phase was slow and then accelerated, showing a final plateau. Four strains were isolated from the compost, and *Aspergillus versicolor* was the best PBS-degrading microorganism.

## 7. Applications

**Figure 12:**
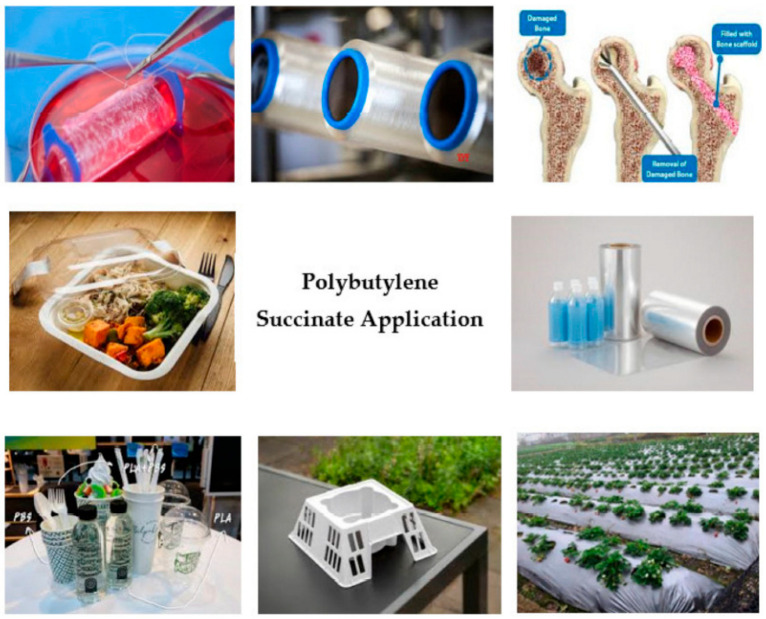
PBS applications [145].

Definitely, PBS, its copolymer with adipic segment, and the blends with PLA, PHBV or TPS have found commercial applications in many fields [144] such as packaging, agriculture, fishery, forestry, construction, and electronics as demonstrated by Figure 12 [145].

For example, PBS and PBS/PLA blends have been employed as mulch films [146,147], packaging [148], and dishware (as stated by Pivsa-Art at al. [149]). In addition, they are used in foaming [150], drug encapsulation systems [151], orthopedic applications [152], coffee capsules [153], and the industrial field [154].

The processability of PBS is related to its molecular weight: a Mw of less than 100,000 is desirable for extrusion and injection molding; with higher Mw or long, branched chains, film blowing and casting can be used [155]. Thermal stability and a high crystallization rate are fundamental to ensure a smooth processing. To increase crystallinity and transparency, nucleating agents can be used [156].

The performance expected from bioplastic materials used in food packaging applications is to protect it from the environment and maintain food quality. In view of possible applications of PBS and PBSA, Siracusa et al. [157] considered films of PBS and PBSA and evaluated the permeability behavior and the changes after contact with foods. From the analysis, no severe damage of the materials was observed, and the results confirmed the potential of these materials for the food industry. Recent work showed the insertion in the market of PBS reinforced with natural plant fibers [158].

## 8. Conclusions

This brief technical review aimed to substantially examine the current main knowledge and prospects of poly (butylene succinate) (PBS) and its copolymers, in particular poly (butylene succinate-co-butylene adipate) (PBSA). Owing to its balanced properties, good thermal processability window, and biodegradability, PBS and its copolymers are attracting much interest in this era of increasing environmental awareness. Furthermore, the properties and biodegradation rate of PBS can be tailored via copolymerization with different types and contents of comonomer units to meet various requirements.

At the present stage, several companies are planning to expand their manufacturing capabilities of PBS to meet the rapid increase in demand for biodegradable plastics, especially for packaging purposes. However, only future developments and a progressive change of mindset will make PBS and other bio-based polymers more and more present in our lives.

## Figures and Tables

**Table 1:** Global producers of PBS (Reprinted with permission from Reference [57] Copyright 2020 Elsevier).

Company	Location	Brand Name	Production (Kton/Year)
BASF	Germany	PBS	not available
Dupont	USA	PBST	not available
Hexing Chemicals	China	PBS	3
IPC-CAS	China	PBS, PBSA	5
IRE Chemical	Korea	PBS, PBSA	3.5
Kingfa	China	PBSA	1
Mitsubishi Gas Chemical	Japan	PBS, PBSA, PES	3
Showa	Japan	PBS, PBSA	3
SK Chemicals	Korea	Skygreen	not available

## Data Availability

Not available.
